# Editorial: Crosslinking of feed nutrients, microbiome and production in ruminants

**DOI:** 10.3389/fvets.2025.1610490

**Published:** 2025-06-17

**Authors:** Wenjuan Du, Wenjie Xu, Yin Hu, Shengguo Zhao, Fuyong Li, Deguang Song, Junshi Shen, Qingbiao Xu

**Affiliations:** ^1^College of Animal Sciences and Technology, Huazhong Agricultural University, Wuhan, China; ^2^Key Laboratory of Quality & Safety Control for Milk and Dairy Products of the Ministry of Agriculture and Rural Affairs, Institute of Animal Sciences, Chinese Academy of Agricultural Sciences, Beijing, China; ^3^Department of Animal Science and Technology, College of Animal Sciences, Zhejiang University, Hangzhou, Zhejiang, China; ^4^Department of Immunobiology, Yale University School of Medicine, New Haven, CT, United States; ^5^Ruminant Nutrition and Feed Engineering Technology Research Center, College of Animal Science and Technology, Nanjing Agricultural University, Nanjing, China

**Keywords:** ruminants, feed nutrients, rumen microbiome, rumen fermentation, interactions

In ruminants, the rumen serves as the primary organ responsible for converting plant-based feed into nutrients and energy. Research indicates that microbial derivatives, dietary composition, and host metabolism collectively affect rumen metabolite concentrations and microbial community structure, shaping the mechanisms of host-microbiota interactions (1). Ruminants rely on symbiotic relationships with complex rumen microbial communities that specialize in degrading recalcitrant plant polymers such as cellulose and hemicellulose, converting them into digestible compounds. These microbes are critical for the productivity and health of ruminants because they directly contribute to volatile fatty acid (VFA) production and microbial protein biosynthesis, both of which are essential to milk efficiency (2). Additionally, bacterial communities influence growth performance as well as milk yield and composition in dairy cattle (3). Conversely, rumen microbiota structure is modulated by host species, dietary energy levels, and environmental factors (4). This Research Topic primarily explores nutritional interventions to regulate growth performance, rumen fermentation, and microbial composition in ruminants.

Diet and feed additives are potent modulators of rumen microbiota, serving as substrates for microbial metabolism and thereby altering rumen environments and species composition. Early dietary interventions may help establish rumen microbial communities, leading to long-term changes in community structure and function that ultimately affect host phenotypes (5). For instance, Liu J. et al. found that supplementing 0.3% moringa polysaccharides to the milk replacer of early-weaned goat kids increased their average daily gain (ADG), feed intake, serum immunoglobulins (IgA and IgM), rumen muscle thickness, rumen wall thickness, and rumen pH, while also enriching *Actinobacteria* and *Butyrivibrio* species in the rumen. Zhang S. et al. reported that adding 1,500 mg/kg of guanidinoacetic acid to the diet increased ruminal ammonia nitrogen concentration and total reducing sugar flow into the small intestine, thereby improving creatine levels, glucose utilization, and average daily gain (ADG) in lambs. Hou et al. demonstrated that supplementing 5% residual black wolfberry fruit enhanced growth performance in Duolang sheep, optimized rumen fermentation parameters without negatively affecting microbial structure, and improved economic returns. Luo et al. found that nisin and monensin supplementation in fattening Hu sheep reduced ruminal acetate concentration and altered fermentation patterns, although it did not affect their growth performance or health.

Studies have suggested that fermented feed products can enhance antioxidant and immune capacity, improve rumen fermentation, and modulate microbial communities in ruminants (6). Cheng et al. reported that 15% fermented rice husk feed improved the growth performance, nutrient digestibility, and ruminal propionate, butyrate, and valerate concentrations of Hu sheep, while enriching fiber-degrading bacteria (e.g., *Ruminococcus*) and suppressing inefficient taxa (e.g., *Rikenellaceae RC9*). Thus, fermented rice husk represents a promising alternative to conventional roughage. Zhang J. et al. observed that fermented soybean meal did not affect milk yield in lactating cows but increased serum prolactin levels and altered rumen microbiota, potentially benefiting long-term health and productivity. Liu Y. et al. demonstrated that dietary fermented jujube powder (FJP) enhanced ADG and feed efficiency by promoting nutrient degradation and VFA production via microbial enzyme activity. Additionally, elevated serum total antioxidant capacity and reduced malondialdehyde levels indicated improved oxidative defense, underscoring FJP's potential as a functional feed additive.

Yeast culture (YC), a feed additive rich in yeast cell wall components (e.g., mannan oligosaccharides, and β-glucans) and fermentation metabolites (e.g., organic acids, B vitamins, and enzymes), stabilizes rumen pH and promotes fiber-degrading bacteria, enhancing feed efficiency (7). Li et al. found that 10 g/d YC supplementation in dairy goats increased milk yield and ruminal acetate, butyrate, and VFA concentrations while reducing NH3-N levels, suggesting improved microbial protein synthesis. Zhang L. et al. reported that *Saccharomyces cerevisiae*-fermented sorghum distillers' grains reduced weight loss in early-lactation goats and improved milk quality, likely via bile acid and caffeine metabolism pathways linked to energy and immune regulation.

Milk, a vital nutrient source for humans, contains lactose, triglycerides, proteins, minerals, and vitamins. β-casein, a major milk protein, exists in two primary genotypes (A1 and A2) that differ at position 67 (histidine in A1, proline in A2). A2 milk (from A2A2 genotype cows) is considered more digestible and health-promoting. Zhao et al. identified unique rumen microbial and metabolic profiles (e.g., arachidonic acid, adrenic acid, glycocholic acid, taurine, and *g_Acetobacter*) in A2A2 cows that correlated with higher milk fat content. Arachidonic acid, a key biomarker, may enhance milk fat synthesis by activating lipogenic genes.

Amino acids (AAs), the building blocks of proteins, are central to protein nutrition. Reducing dietary protein levels while supplementing limiting AAs (e.g., lysine and methionine) can meet ruminant requirements. Wang et al. showed that a 3: 1 ratio of rumen-protected Lys and Met in Holstein bulls improved nitrogen efficiency and stabilized rumen microbiota without compromising intake or digestibility, offering a strategy for stress-resistant feeding. L-carnosine, a dipeptide with antioxidant and anti-inflammatory properties, was shown by Meng et al. to enhance growth performance in fattening lambs by modulating gut microbiota and serum metabolites, thereby promoting protein synthesis and energy metabolism.

Alfalfa hay, a widely used forage in livestock production due to its high palatability, low fiber content, and high protein content (17%−22%), exhibits nutrient variability depending on cultivar, storage method, and harvest stage, which may influence dairy cow performance and rumen microbiota. La et al. observed that while alfalfa hay from different sources (Spanish SAH vs. American AAH) significantly altered the rumen microbial composition and function of dairy cows, these changes did not affect their production performance, nutrient digestibility, or blood biochemical parameters. This suggested SAH as a viable alternative to mitigate market supply fluctuations while maintaining productivity.

As the demand for high-quality dairy and beef rises, Holstein cattle—a globally dominant dairy breed—have been extensively studied, whereas indigenous breeds such as Chinese Sanhe cattle (which are dual-purpose for milk and meat production) remain under-researched. Liu Z. et al. compared rumen microbiota between multiparous Sanhe and Holstein cows, finding similar species compositions although there were variations in abundance by parity and breed. Rumen ecology was found to strongly correlate with metabolic patterns; however, breed remained the decisive factor for productivity. Crossbreeding, a strategy to enhance growth and feed efficiency, introduces superior traits into local breeds. Zhang R. et al. demonstrated that crossbreeding alters rumen microbiota and metabolites, significantly improving growth. ♂Poll Dorset × ♀Hu crosses were found to enhance fiber fermentation and energy supply, while ♂Southdown × ♀Hu crosses optimize amino acid metabolism for protein synthesis, providing insights for breeding and nutrition strategies.

In summary, ruminant digestion relies on an intricate host-microbe symbiosis, where feed nutrients shape microbial activity, influencing nutrient absorption and production outcomes. Future research should focus on precise microbiota modulation, functional feed development, and sustainable farming to enhance productivity, product quality, and environmental stewardship. These advances will revolutionize ruminant production systems.
